# Dual Channel S-Band Frequency Modulated Continuous Wave Through-Wall Radar Imaging

**DOI:** 10.3390/s18010311

**Published:** 2018-01-22

**Authors:** Ying-Chun Li, Daegun Oh, Sunwoo Kim, Jong-Wha Chong

**Affiliations:** 1 Department of Electronic Engineering, Hanyang University, Seoul 04763, Korea; davis.y.lee@hotmail.com (Y.-C.L.); remero@hanyang.ac.kr (S.K.); 2 Collaborative Robots Research Center, Daegu Gyeongbuk Institute of Science and Technology, Daegu 42988, Korea; dgoh@dgist.ac.kr

**Keywords:** 3D shift invariant, TWRI, FMCW, dual channel

## Abstract

This article deals with the development of a dual channel S-Band frequency-modulated continuous wave (FMCW) system for a through-the-wall imaging (TWRI) system. Most existing TWRI systems using FMCW were developed for synthetic aperture radar (SAR) which has many drawbacks such as the need for several antenna elements and movement of the system. Our implemented TWRI system comprises a transmitting antenna and two receiving antennas, resulting in a significant reduction of the number of antenna elements. Moreover, a proposed algorithm for range-angle-Doppler 3D estimation based on a 3D shift invariant structure is utilized in our implemented dual channel S-band FMCW TWRI system. Indoor and outdoor experiments were conducted to image the scene beyond a wall for water targets and person targets, respectively. The experimental results demonstrate that high-quality imaging can be achieved under both experimental scenarios.

## 1. Introduction

Through-the-wall radar (TWR) techniques have been investigated in the past few years as a way to support police and soldiers operating in urban areas (e.g., when breaking into a room occupied by hostile agents) and to help first responders in search and rescue operations (e.g., when looking for people trapped inside buildings on fire or buried under rubble).

When the transmitted TWR signal encounters a wall, most of the transmitted signals are reflected due to the short wavelength of the transmitted signal in comparison with the dimensions of the wall. In TWR systems, the reflected signals from the wall are much stronger than those from the targets behind the wall. The presence of strong undesired signals limits the dynamic range of TWR systems and increases the possibility of saturation and blocking of the receiver [1]. Furthermore, it has been observed that this prevents the application of compressive sensing techniques, which aim at producing radar images of the same quality and resolution using far fewer data, thus allowing faster measurements [2].

In recent decades, TWR systems have been used to detect and track targets behind walls by estimating the distance and velocity of targets [3,4]. However, especially in rescue missions, surveillance, and reconnaissance, the exact locations of targets are urgently needed. Thus, through-the-wall radar imaging (TWRI) has been recently sought out to image the relative position, illustrated by down-range and cross-range, in a certain coordinate system [5,6,7].

There are several waveforms utilized in TWRI systems, including pseudo-noise waveforms based on M-sequences [8], stepped-frequency continuous wave (SFCW) signals [9,10,11], and frequency-modulated continuous wave (FMCW) signals [12,13,14,15,16].

Several prototypes of TWRI systems using UWB impulses have been developed [17,18,19] due to their high resolution in time delay estimation. However, UWB impulse radar is very complex, especially in the design of signal processing platforms, due to the high sampling rate of ADC which is proportional to signal bandwidth. In contrast, even if the bandwidth of FMCW radar is the same as that of UWB pulse radar, FMCW radar can be implemented at low cost due to its low sampling rate as compared with that of UWB pulse radar.

The FMCW radar is a cost effective alternative to high resolution radar. Because of their low complexity and low sampling rates, FMCW radars are inexpensive and easy to manufacture, relatively free from failure, and cheap to maintain. Thus, the FMCW technique was adopted in this article.

Among the many interesting aspects of TWRI, such as wall parameter estimation [20], wall clutter mitigation [5] and compensation of the angle estimations due to the wall [6], our research interest is only focused on target imaging behind walls.

Most of the TWRI systems [1,21,22] using FMCW signals adopt synthetic aperture radar (SAR) signal processing, which requires a movement of the radar and a huge amount of data processing. Thus, even though SAR yields a high quality TWRI, SAR-based TWRI systems may not be suitable in situations in which information regarding what is behind walls must be obtained rapidly, i.e., terror attacks, military purposes, rescue situations, etc.

A low complexity TWRI system using FMCW signals is presented in this article. Our developed TWRI system is equipped with one transmitting antenna, *Tx*, and two receiving antennas, *Rx*. Based on this two-receiving-antenna structure, we proposed an optimized dual channel TWRI algorithm, which exploits a 3D shift-invariant structure in 3D range-angle-Doppler subspace, resulting in high resolution images of targets. Compared with previously developed TWRI systems, our TWRI system is realized with low complexity, using only two channels. Finally, experimental trials were conducted, and the results demonstrated the high performance of our developed low complexity TWRI system and the proposed high resolution subspace algorithm.

## 2. System Model

Prior to introducing the proposed algorithm for low complexity TWRI, the fundamentals of the system model for TWRI and the simplified signal model for the algorithm are presented, respectively, in this section.

**Figure 1:**
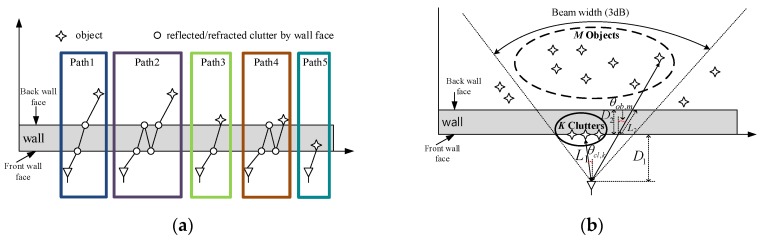
Geometry of TWRI system, (**a**) practical models of wave propagation, (**b**) simplified model for proposed algorithm.

In the TWRI system literature [6,23,24,25,26], a variety of wave propagation models have been studied. Summarizing the suggested models in previous articles, five propagation models are described in Figure 1a, including reflection, refraction and wall ringing within a wall. Path 1 and path 2 show that a wave penetrates the wall and is reflected by an object behind wall, accompanied by refraction in path 1 and wall ringing within the wall in path 2. In contrast, paths 3 to 5 are caused by clutters on front/back face of the wall. As a simplification of practical signal model for TWRI systems introduced in [26], we adopt a simplified signal model including paths 1 and 5 in this paper as shown in Figure 1b. The transmitted signal is propagated along the direct path between an antenna and an object, without consideration of the refraction on the interface between the wall and air. Meanwhile, for the undesired clutter signal we only focus on that reflected by the front face of the wall.

Related to TWRI, a lot of research areas have been explored in previous works, including wall parameter estimation [20], compensation for refraction due to the wall [6], SAR for high resolution [22], and so far. Since we focused on presenting a low complexity TWRI algorithm, the other research topics related to TWRI are not addressed in this paper. 

Let us denote the number of wall clutters and the number of the objects behind walls in the field of view, *K* and M, respectively, as shown in Figure 1b. 

The round-trip delay (RTD) of the *k*-th clutters *τ_cl,k_* can be obtained by:

(1) $$\tau_{cl,}{}_{k}=2\frac{L_{1}}{c}=2\frac{D_{1}}{c\cdot \cos\theta_{cl,k}}$$

where *L*_1_ is the standoff distance between the antenna and clutters at the front face of the wall, *c* is the speed of light in free space, and *θ_cl,k_* is the direction of arrival (DOA) of received signal from *k*-th clutter.

Assuming that the *m*-th object at the direct path distance *R_m_* away from the antenna is moving with a constant radial velocity *v_m_* over *P* pulses, the direct path distance between the antenna and the *m*-th object for the *p*-th pulse is changed to:

*R_p,m_* = *R_m_* + *v_m_* × *pT_PRI_* (2)

where *T_PRI_* is the pulse repetition interval (PRI). Thus, the RTD of the *m*-th object for the *p*-th pulse can be obtained as in [26] such that:

(3) $$\tau_{ob,p,m}=2\left(\frac{R_{p,m}}{c}+\frac{L_{2}}{c}\left(\sqrt{\varepsilon }-1\right)\right)=2\left(\frac{R_{p,m}}{c}+\frac{D_{2}}{c\cdot \cos\theta_{ob,m}}\left(\sqrt{\varepsilon }-1\right)\right)$$

where *D* is the thickness of the wall, and *ε* is the dielectric constant of the wall. It can be easily derived that *τ_ob,p,m_* ≫ *τ_cl,k_* in proportional to *D* and *ε* by comparing *τ_ob,p,m_* of Equation (3) with *τ_cl,k_* of Equation (1).

The transmitted *P* FMCW chirp pulses can be modeled by:

(4) $$\begin{array}{l} s_{pulses}(t)=\sum_{p=0}^{P-1}s\left(t-pT_{PRI}\right)\\ \mathrm{where}\;s(t)=\{\begin{array}{l} \exp\left[j\left(2\pi f_{c}t+\frac{\mu }{2}t^{2}\right)\right]\;\mathrm{for}\;0\leq t<T_{sym}\\ 0\;\mathrm{elsewhere} \end{array} \end{array}$$

*f_c_* denotes the carrier frequency, *μ* is the rate of change of the instantaneous frequency of a chirp signal, and *T_sym_* is the duration of the FMCW chirp pulse. The bandwidth of the FMCW chirp signal is defined by *f_BW_* = *μT_sym_*/2π. 

Considering two receiving antennas, separated by a distance *d*, the received *p*-th pulse at the *l*-th antenna can be represented from the TWRI simplification of [26] by:

(5) $$\begin{array}{l} r_{p,l}(t)=\sum_{k=0}^{K-1}a_{cl,k}\exp\left(j\frac{\pi }{\lambda_{s}}dl\sin\theta_{cl,k}\right)s\left(t-\tau_{cl,k}\right)+\\ \sum_{m=0}^{M-1}a_{ob,m}\exp\left(j\frac{\pi }{\lambda_{s}}dl\sin\theta_{ob,m}\right)s\left(t-\tau_{ob,p,m}\right) \end{array}$$

where *a_cl,k_* and *a_ob,m_* denote the complex amplitude for the *k*-th clutter and the *m*-th object, respectively, and *λ_s_* denotes the wavelength of the carrier signal, for *l* = 0 and 1. 

**Figure 2:**
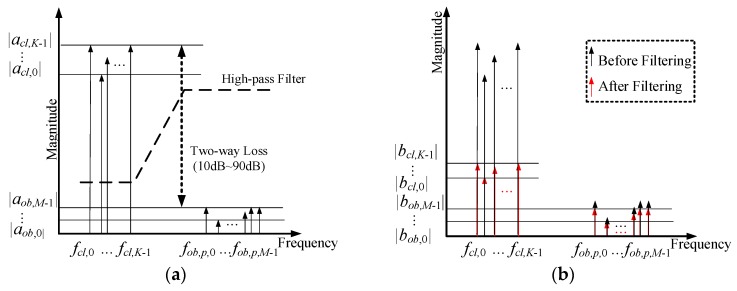
Illustration of high-pass filtering, (**a**) Beat signals before filtering, (**b**) Filtered beat signals.

In an FMCW radar, received chirp signals can be easily transformed into a sinusoidal waveform by de-chirping, which involves multiplying the received signal by the transmitted chirp replica, followed by low-pass filtering [16], as shown in Figure 2b. We call these sinusoids beat signals. The *p*-th beat signal at the *l*-th antenna can be represented as in [16] by:

(6) $$y_{p,l}\left(t\right)=r_{p,l}\left(t\right)\times s\left(t\right)=b_{cl}(t)+b_{ob}(t)\;\mathrm{where}\;$$

(7) $$b_{cl}(t)=\sum_{k=0}^{K-1}a_{cl,k}\exp\left(j\frac{\pi }{\lambda_{s}}dl\sin\theta_{cl,k}\right)\exp\left(j\left(f_{cl,k}t+2\pi f_{c}\tau_{cl,k}-\frac{\mu }{2}\tau_{cl,k}{}^{2}\right)\right)$$

(8) $$b_{ob}(t)=\sum_{m=0}^{M-1}a_{ob,m}\exp\left(j\frac{\pi }{\lambda_{s}}dl\sin\theta_{ob,m}\right)\exp\left(j\left(f_{ob,p,m}t+2\pi f_{c}\tau_{ob,p,m}-\frac{\mu }{2}\tau_{ob,p,m}{}^{2}\right)\right)$$

where *f_cl_*_,*k*_ and *f_ob_*_,*p*,*m*_ are the frequencies of the transformed beat signals from the wall clutter and object, respectively, by:

(9) $$f_{cl,k}=\mu \tau_{cl,k}\;\mathrm{and}\;f_{ob,p,m}=\mu \tau_{ob,p,m}$$

In a through-wall radar imaging scenario, the speed of the objects *v* is assumed to be much smaller than that of vehicles. Thus, the effects of the movement over *P* pulses on the complex amplitude and DOA can be ignored as in Equation (3), Equation (5), and Equation (8). We only considered it in RTD as in Equation (8). 

Substituting *τ_ob,p,m_* with *R_p_*_,*m*_ of Equation (2) in Equation (8), the beat signals from an object behinds wall can be modeled with some approximation of [27], by

(10) $$b_{ob}(t)\approx \left(\sum_{m=0}^{M-1}\begin{array}{l} a_{ob,m}\exp\left(j\frac{\pi }{\lambda_{s}}dl\sin\theta_{ob,m}\right)\\ \times \exp\left(j\left(2\pi f_{c}\frac{2(R_{m}+v_{m}pT_{PRI})}{c}+\left(\frac{2\mu R_{m}}{c}\right)t+\eta_{m}\right)\right) \end{array}\right)$$

where *η_m_* denotes the fixed phase term for the *m*-th object, induced in the process of approximation. 

Generally, the two-way loss due to a wall can be represented by

(11) $$TwoWayLoss\left(dB\right)\approx 10\log10\left(\left|\frac{a_{ob,m}}{a_{cl,k}}\right|\right)$$

and the loss is determined by the material and thickness of the wall. In the case of a reinforced concrete wall, a 20 cm thickness yields 90 dB two way loss as in [16]. From the relationship of *τ_ob,p,m_* ≫ *τ_cl,k_*, the frequency of the transformed beat signals also can be related to each other, such that, *f_ob,p_*_,*m*_ ≫ *f_cl_*_,*k*_. If the frequencies for clutters and objects can be grouped separately from each other because of the wall, as shown in Figure 2a, a low pass filter can be applied with the beat signals to reduce the gap between |*a_cl,k_*| and |*a_ob,m_*|, as illustrated in Figure 2. If the gap is not reduced in the specific case of reinforced concrete wall of 20 cm, most of the dynamic range of ADC is fit to the magnitude level of wall clutter, |*a_cl,k_*|. This causes the SNR for the digitized samples for the beat signals from the *M* objects to be very poor due to two-way loss, leading to missed detection. Thus, most conventional FMCW through wall radar systems make use of a high-pass filter to reject the wall-induced components. 

The magnitudes |*a_cl,k_*| and |*a_ob,m_*| in Figure 2a are replaced with |*b_cl,k_*| and |*b_ob,m_*| in Figure 2b to describe the filtered beat signals. Since a high-pass filter is used for wall clutter rejection, |*a_cl,k_*| ≫ |*b_cl,k_*| and |*a_ob,m_*|≈|*b_ob,m_*|.

After analog-to-digital conversion, the discrete time model for *r_p_*_,*l*_(*t*) of Equation (5) with the sampling frequency *f_s_* = 1/*T_s_* satisfying the Nyquist criterion can be derived by *y_p,l_* [*n*] = *y_l_* (*nT_s_*) for *p* = 0,…, *P* − 1, *l* = 0 and 1, and *n* = 0,..., *N* − 1, where *N* = *T_sym_*/*T_s_*.

## 3. Conventional FMCW TWRI Systems

In previous works [28,29,30,31,32,33], many methods and architectures have been proposed for TWRI systems based on SFCW or FMCW signals, as well as synthetic aperture radar (SAR) principles. These conventional works on TWRI systems are briefly summarized in this section.

### 3.1. Subspace Projection Approach

In [28], the signals received by the *M* objects are arranged into a *Q* × *L* matrix:

(12) $$Z=\left[z_{0}\;\cdots \;z_{l}\;\cdots \;z_{L-1}\right],\mathrm{where}\;z_{l}=\left[z_{l}(f_{cl,0})\;\cdots \;z_{l}(f_{cl,K-1})\;z_{l}(f_{ob,p,0})\cdots \;z_{l}(f_{ob,p,M-1})\right]$$

The eigenstructure of the imaged scene is obtained by performing the SVD of ***Z***:

(13) $$Z=U\Lambda V^{H}$$

where *H* denotes the Hermitian transpose, ***U*** and ***V*** are unitary matrices containing the left and right singular vectors, respectively, Λ is a diagonal matrix:

(14) $$\Lambda =\left[\begin{array}{ccc} \lambda_{1} & \cdots & 0\\ \vdots & \ddots & \vdots \\ 0 & \cdots & \lambda_{L}\\ \vdots & \ddots & \vdots \\ 0 & \cdots & 0 \end{array}\right]$$

and $\lambda_{1}\geq \lambda_{2}\geq \cdots \geq \lambda_{L}$ are the singular values. The subspace projection method assumes that the wall returns and the target reflections lie in different subspaces. Since the wall reflections are stronger than the target returns, the first *J* dominant singular vectors of the ***Z*** matrix are used to construct the wall subspace [29].

### 3.2. SAR-Based Approach

In [30,31,32,33], the SAR principle is used to extend standard radar imaging to account for the presence of the wall in the TWRI system. The novel combination of FMCW technology and SAR techniques leads to the development of a small, lightweight, and cost-effective high resolution imaging sensor. In [30], an algorithm for processing FMCW SAR signals is presented; however, it requires the complete bandwidth of the transmitted signal to be sampled and a single long FFT to be performed over all of the collected data for the processing. FMCW radar technology is of interest for both civil and military airborne Earth observation applications, especially in combination with high resolution SAR techniques.

**Figure 3:**
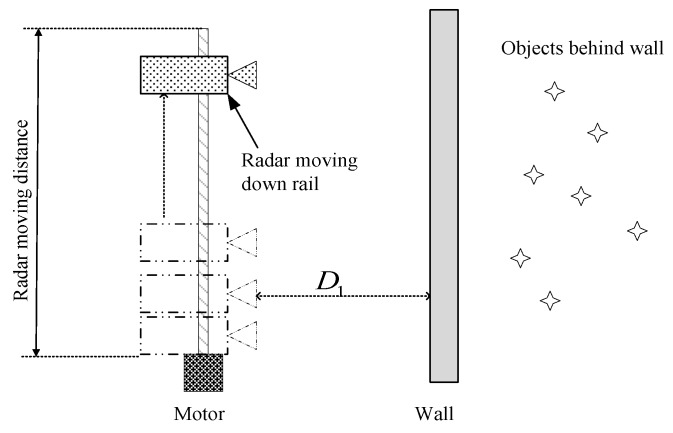
TWRI SAR geometry in [31].

In Figure 3, a typical SAR-based TWRI geometry, in which FMCW radar is mounted on a moving platform equipped with a motor, is presented from [31].

This kind of SAR-based TWRI system is not suitable in emergency situations such as terror attacks and rescue missions because the entire SAR system cannot be conveniently transported. Moreover, conventional SAR-based TWRI systems [31,32,33] are developed with many antennas for the realization of high quality imaging.

Unlike the conventional SAR-based FMCW systems with several antennas for high quality imaging, in this paper, a dual channel S-band FMCW TWRI system is proposed for low complexity realization and high mobility.

## 4. 3D Subspace-Based TWRI Algorithm

For high resolution TWRI with two receiving antennas without movement for the SAR approach, the 3D subspace-based algorithm is presented with the beat samples, *y_p,l_*[*n*] = *y_p_*_,*l*_(*nT_s_*) for *p* = 0,…, *P* − 1, *l* = 0 and 1, and *n* = 0,..., *N* − 1. The main principle of the propose method is related to the 3D shift invariant structure of the matrix of Equation (18), stacked in column direction.

### 4.1. Phase Shift Characteristics

Before describing the principle of the proposed method, we present the phase relationships of the beat samples *y_p,l_* [*n*] between the adjacent samples, the adjacent pulses, and the adjacent antennas, in association with range, Doppler and angle, respectively. For the convenience of describing the phase shifts without loss of generality, we assume a single object environment, i.e., *K* = 0 and *M* = 1 in Equation (6). In this condition where no wall clutter is assumed, *y_p,l_*[*n*] = *b_ob_*(*nT_s_*) from Equations (6)–(8). Then, three kinds of the phase shifts, *κ_m_*, *ξ_m_* and *ρ_m_* can be defined from the approximated model for *b_ob_*(*nT_s_*) in Equation (10) as follows:

- Range-induced phase shift *κ_m_*:
  (15) $$\begin{array}{ll} \kappa_{m} & =y_{p,l}\left[n+1\right]\times {\left(y_{p,l}\left[n\right]\right)}^{*}\;\\ & ={\left|a_{ob,m}\right|}^{2}\exp\left(j\left(\left(\frac{2\mu R_{m}}{c}\right)T_{s}\right)\right) \end{array}$$
- DOA-induced phase shift *ξ_m_*:
  (16) $$\begin{array}{ll} \xi_{m} & =y_{p,l+1}\left[n\right]\times {\left(y_{p,l}\left[n\right]\right)}^{*}\;\\ & ={\left|a_{ob,m}\right|}^{2}\exp\left(j\frac{\pi }{\lambda_{s}}d\sin\theta_{ob,m}\right) \end{array}$$
- Doppler-induced phase shift *ρ_m_*:
  (17) $$\begin{array}{ll} \rho_{m} & =y_{p+1,l}\left[n\right]\times {\left(y_{p,l}\left[n\right]\right)}^{*}\;\\ & ={\left|a_{ob,m}\right|}^{2}\exp\left(j\left(\frac{4\pi f_{c}T_{PRI}}{c}v_{m}\right)\right) \end{array}$$

### 4.2. 3D Smoothed Hankel Matrix

Since the goal of the proposed method is to estimate range, angle and Doppler jointly, the 3D shift invariant matrix is employed for 3D subspace-based processing. 

By stacking the Hankel matrices in a specific way, the 3D shift invariant structure is satisfied for the sample, pulse, and antenna domain, respectively. 

Prior to introducing the 3D shift invariant structure, a 1D shift invariant structure realized by a 1D smoothed Hankel matrix is demonstrated as follows. 

For *y_p,l_*[*n*], *n* = 0,…, *N* − 1, *l* = 0, 1, the single smoothed Hankel matrix ***Y****_p_*_,*l*_ can be made as:

(18) $$Y_{p,l}=\left[\begin{array}{cccc} y_{p,l}\left[0\right] & y_{p,l}\left[1\right] & \cdots & y_{p,l}\left[L_{r}-1\right]\\ y_{p,l}\left[1\right] & y_{p,l}\left[2\right] & \cdots & y_{p,l}\left[L_{r}\right]\\ \vdots & \vdots & \vdots & \vdots \\ y_{p,l}\left[L_{c}-1\right] & y_{p,l}\left[L_{c}\right] & \cdots & y_{p,l}\left[N-1\right] \end{array}\right]\in {\mathbb{C}}^{L_{c},L_{r}},$$

where ${\mathbb{C}}^{a,b}$ denotes the complex matrix space of *a* by *b*, and *L_r_* and *L_c_* = *N* − *L_r_* + 1 are the selection parameters, referred to as pencil parameter in [34], which satisfy the conditions *L_r_* ≥ *M* and *L_c_* ≥ *M*. Shifting the Hankel matrix in Equation (18) up (dropping the first row) or down (dropping the last row) results in a matrix with a row space that lies within the row space of the original matrix, which indicates that the row space of the matrix in Equation (18) is a shift-invariant subspace. 

A 2D smoothed matrix ***Y****_l_* is obtained by stacking single smoothed Hankel matrices ***Y****_p_*_,*l*_:

(19) $$Y_{l}=\left[\begin{array}{c} Y_{0,l}\\ Y_{1,l}\\ \vdots \\ Y_{P-1,l} \end{array}\right]$$

Shifting the matrix in Equation (19) up (dropping the first matrix ***Y***_0,*l*_) or down (dropping the last matrix ***Y****_P_*_−1,*l*_) results in a matrix with a row space that lies within the row space of the original matrix in Equation (19), which indicates that the row space of the matrix in Equation (19) is also a shift-invariant subspace.

Obviously, matrices ***Y****_l_* share one identical row space. Namely, for the matrix formed by stacking ***Y****_l_* column, when *l =* 0, 1,.., *L* − 1, dropping the first segment ***Y***_0_ or dropping the last matrix ***Y****_L_*_−1_ results in a matrix with a row space that lies within the row space of the original matrix. In our proposed FMCW TWRI system, the number of receive antennas is two, which makes for a special 3D smoothed matrix case with only two segments:

(20) $$Y=\left[\begin{array}{c} Y_{0}\\ Y_{1} \end{array}\right]$$

**Figure 4:**
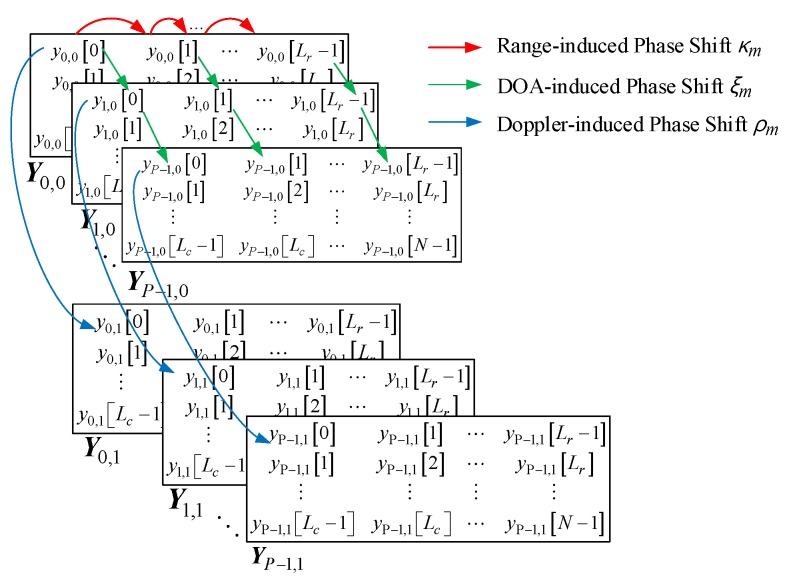
3D Shift Invariant Structure.

Conventional subspace-based algorithms such as Matrix Pencil [34] make use of a 1D Shift Structure to obtain the frequency estimation. Like conventional algorithms, three frequency parameters *κ_m_*, *ξ_m_* and *ρ_m_* need to be estimated in our proposed FMCW TWRI system; a 3D Shift Invariant Structure is proposed, which is formed by organizing a 3D smoothed Hankel matrix, as shown in Figure 4. 

### 4.3. Subspace Separation with SVD

The matrix ***Y*** in Equation (20) is factored by singular value decomposition (SVD) such that:

(21) $$Y=U\Sigma V^{H},\;\mathrm{where}\;U=\left[\begin{array}{cc} U_{s} & U_{n} \end{array}\right],\Sigma =\left[\begin{array}{cc} \Sigma_{s} & \Sigma_{n} \end{array}\right]\;\mathrm{and}\;V=\left[\begin{array}{cc} V_{s} & V_{n} \end{array}\right]$$

In Equation (21), the submatrices ***U****_s_*, ***Σ****_s_*, and ***V****_s_* are associated with the signal subspace, and the submatrices ***U****_s_*, ***Σ****_s_*, and ***V****_s_* are associated with the noise subspace. The signal subspace and noise subspace are defined by:

(22) $$Y=\underset{\mathrm{signal}\;\mathrm{subspace}}{\underset{︸}{U_{s}\Sigma_{s}V_{s}{}^{H}}}+\underset{\mathrm{noise}\;\mathrm{subspace}}{\underset{︸}{U_{n}\Sigma_{n}V_{n}^{H}}}$$

After SVD on ***Y***, the matrices ***U***, ***Σ***, and ***V*** are given as a pair. However, the separation of Equation (22) is not automatically supported by SVD; the derived singular values must be used in a specific manner. The criterion of the minimum description length (MDL) [35] is adopted in this paper for classification of the signal and noise subspaces shown in Equation (22).

Assuming noiseless data for convenience, we have:

(23) $$Y=U_{s}\Sigma_{s}V_{s}{}^{H}$$

For smoothed Hankel matrix ***Y****_p_*_,*l*_ a steering matrix ***A***_1*D*_ with Vandermonde structure is defined in terms of the components of the M sinusoids of *y_p,l_*(*t*) by:

(24) $$A_{1D}=\;\left[\begin{array}{cccc} 1 & 1 & \cdots & 1\\ \kappa_{0} & \kappa_{1} & \cdots & \kappa_{M-1}\\ \vdots & \vdots & \ddots & \vdots \\ \kappa_{0}^{L_{c}-1} & \kappa_{1}^{L_{c}-1} & \cdots & \kappa_{M-1}^{L_{c}-1} \end{array}\right]\in {\mathbb{C}}^{L_{c},M}$$

Assuming a noiseless matrix ***Y****_p_*_,*l*_ for convenience, a basis for the column space for ***Y****_p_*_,*l*_ of rank *M* can be defined as the set of columns in ***A*** since the phase shift *κ_m_* corresponds to the phase shift of *y_p,l_*(*t*). Thus, there exists a transformation matrix ***F*** of *M* by *L_r_* satisfying ***Y****_p_*_,*l*_ = ***A***_1*D*_***F*** as in [36]. Similarly, for 2D stacked matrix ***Y****_l_* we have ***Y****_l_ =* ***A***_2*D*,*l*_***F***, and ***A***_2*D*,*l*_ is defined as:

(25) $$A_{2D,l}=\left[\begin{array}{c} A_{1D}R_{0,l}\\ A_{1D}R_{1,l}\\ \vdots \\ A_{1D}R_{P-1,l} \end{array}\right]=\left[\begin{array}{c} A_{1D}R_{0,l}\\ A_{1D}R_{0,l}\Pi \\ \vdots \\ A_{1D}R_{0,l}\Pi^{P-1} \end{array}\right]\in {\mathbb{C}}^{P*L_{c},M}$$

where:

$$\begin{array}{l} R_{p,l}=diag\left[{\tilde{a}}_{ob,0}\xi_{0}^{l}\rho_{0}^{p}\varsigma_{0},\;{\tilde{a}}_{ob,1}\xi_{1}^{l}\rho_{1}^{p}\varsigma_{1},...,\;{\tilde{a}}_{ob,M-1}\xi_{M-1}^{l}\rho_{M-1}^{p}\varsigma_{M-1}\right]\\ {\tilde{a}}_{ob,m}=a_{ob,m}/\left({\left|a_{ob,m}\right|}^{2L_{c}-2+2l+2p}\right)\mathrm{and}\;\varsigma_{m}=\exp\left(j\left(\frac{4\pi f_{c}R_{m}}{c}+\eta_{m}\right)\right)\\ \Pi =diag[\rho_{0},\rho_{1,...},\rho_{M-1}] \end{array}$$

and *diag*[·] denotes the diagonal matrix.

Finally, the 3D stacked matrix ***Y*** is represented as:

(26) $$Y=A_{3D}F=\left[\begin{array}{c} A_{2D,0}\\ A_{2D,1} \end{array}\right]F=\left[\begin{array}{c} \begin{array}{c} A_{1D}R_{0,0}\\ A_{1D}R_{0,0}\Pi \\ \vdots \\ A_{1D}R_{0,0}\Pi^{P-1} \end{array}\\ \begin{array}{c} A_{1D}R_{0,0}\Psi \\ A_{1D}R_{0,0}\Pi \Psi \\ \vdots \\ A_{1D}R_{0,0}\Pi^{P-1}\Psi \end{array} \end{array}\right]F\in {\mathbb{C}}^{P*L_{c},L_{r}}$$

where $\Psi =diag[\xi_{0},\xi_{1,...},\xi_{M-1}].$

Assuming that the estimated signal subspace ***U****_s_* is correctly separated from the noise subspace, there can be the following relationship between ***A***_3*D*_ of Equation (26) and ***U****_s_*, such that:

(27) $$U_{s}=A_{3D}T^{-1}$$

where ***T*** is an *M* by *M* non-singular transformation matrix, as in [36]. Since *M* objects are assumed in Equation (5), ***U****_s_*, the spanning signal subspace, also has *M* basis vectors. Thus, the steering matrix ***A***_3*D*_, which is composed of *M* column vectors as in Equation (26), is related to ***U****_s_* by Equation (27).

### 4.4. 3D Pseudo-Spectrum for TWRI

Since the signal subspace and noise subspace are orthogonal, we propose a 3D pseudo-spectrum estimation for three kinds of phase shifts, *κ_m_*, *ξ_m_* and *ρ_m_*. 

Assuming three pseudo-spectrum steering vectors are defined such that:

(28) $$\begin{array}{l} s_{q}={\left[\begin{array}{cccc} 1 & \exp\left(j\frac{2\pi }{Q}q\right) & \cdots & \exp\left(j\frac{2\pi }{Q}\left(L_{r}-1\right)q\right) \end{array}\right]}_{1\times L_{r}}\\ s_{w}={\left[\begin{array}{cc} 1 & \exp\left(j\frac{2\pi }{W}w\right) \end{array}\right]}_{1\times L}\\ s_{g}={\left[\begin{array}{cccc} 1 & \exp\left(j\frac{2\pi }{G}g\right) & \cdots & \exp\left(j\frac{2\pi }{G}\left(P-1\right)g\right) \end{array}\right]}_{1\times P} \end{array}$$

for *q* = 0,…, *Q* − 1, *w* = 0,…, *W* − 1, and *g* = 0,…, *G* − 1, respectively.

The 3*D* pseudo-spectrum can be obtained through the vector ***s*** (*q*, *w*, *g*) and the signal subspace spanning matrix ***U****_s_* in Equation (23), such as:

(29) $$Pseudo\left(q,w,g\right)=\frac{1}{{\left[s\left(q,w,g\right)\right]}^{H}U_{s}U_{s}^{H}\left[s\left(q,w,g\right)\right]}$$

where ***s***(*q*,*w*,*g*) = ***s****_q_*⊗***s****_w_*⊗***s****_g_*, and ⊗ denotes the Kronecker product.

By the peak detection method, the *M* peaks can be detected for each 1D pseudo-spectrum searching, and the estimated three indexes ${\left\{q_{m}\right\}}_{m=0}^{M-1},{\left\{w_{m}\right\}}_{m=0}^{M-1},\;{\left\{g_{m}\right\}}_{m=0}^{M-1}$ at which the *M* peaks are found, such that:

(30) $$\left\{q_{m},w_{m},g_{m}\right\}={\left\{\max_{m}\left[Pseudo\left(q,w,g\right)\right]\right\}}_{m=0}^{M-1}$$

where max*_m_*[•] denotes the m-*th* biggest value. Since the three indexes of 3D pseudo-spectrum are estimated, estimations for the ranges, DOAs and velocities of *M* objects behind the wall, can be organized by the relationship in *κm*, *ξ_m_* and *ρ_m_* in Equations (15)–(17), for *m* = 0,…, *M* − 1, respectively:

(31) $${\hat{R}}_{m}=\frac{c}{2\mu }\left(\frac{q_{m}}{Q}\times \frac{1}{T_{s}}\right)$$

(32) $${\hat{\theta }}_{ob,m}=\arcsin\left(\frac{\lambda_{s}}{\pi d}\times \frac{w_{m}}{W}\right)$$

(33) $$\mathrm{and}\;{\hat{v}}_{m}=\frac{c}{4\pi f_{c}T_{PRI}}\times \frac{g_{m}}{G}$$

## 5. Experiments

A concise and precise description of the experimental results will be presented. This section is divided into two subsections.

### 5.1. Experiment Setup

**Figure 5:**
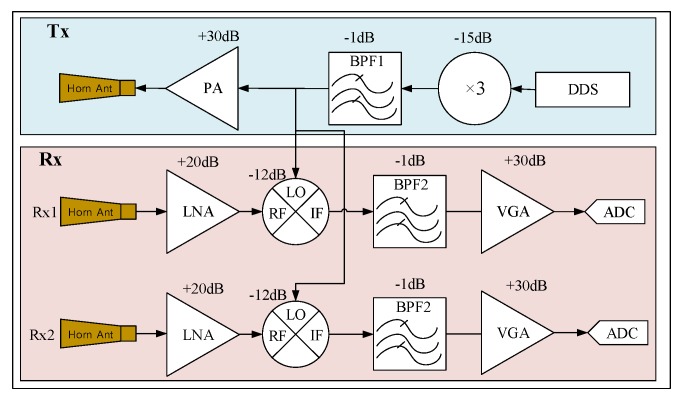
Block diagram of the S-Band FMCW TWRI radar system.

We implemented the proposed S-band FMCW TWRI radar system at 2.9 GHz, which has two receiving antennas and 2 ADC channels. As illustrated in Figure 5, the beat signals from the two ADC channels, which are connected to the two receiving antennas, respectively, were used with the proposed method and the conventional algorithms for imaging.

The transmitter constitutes of an AD9914 Direct Digital Synthesizer (DDS) from Analog Devices (Norwood, MA, USA), a Band Pass Filter (BPF), a frequency multiplier with a multiplication factor of 3, and a Power Amplifier (PA). The AD9914 generates the FMCW source, sweeping over the range from 867 MHz to 1067 MHz, that is, a 200 MHz bandwidth. Through the frequency tripler, the transmitted signal of 2.6 GHz to 3.2 GHz with bandwidth *B* = 600 MHz is obtained. Following the frequency tripler, BPF1, with a frequency range 2.6 GHz to 3.2 GHz is employed to optimize the generated *Tx* signal. In our TWRI system, the other parameters are set as follows: *T_PRI_* = 200 μs, *f_c_* = 2.9 GHz, *f_s_* = 12.5 MHz, *N* = 2048, *P* = 10, *L_c_* = 1000, and *L_r_* = 1049.

The receiver comprises two LNAs, two mixers, two BPFs, two VGAs and two 12-bit ADCs. For each *Rx* channel, one of LNAs will amplify the RF signal with a gain of 20 dB. The amplified RF signal is downconverted to an IF signal (beat signal) by the mixer with a gain of −12 dB. Due to the frequency components induced by the wall, BPF2 is utilized to inject those frequency components, as stated in Section 2. The measured 3 dB cutoff frequency of the BPFs is about 40 KHz. In the implemented system, we sampled the received signal at 13.3 MHz. The gain of single modules are shown in Figure 5.

**Figure 6:**
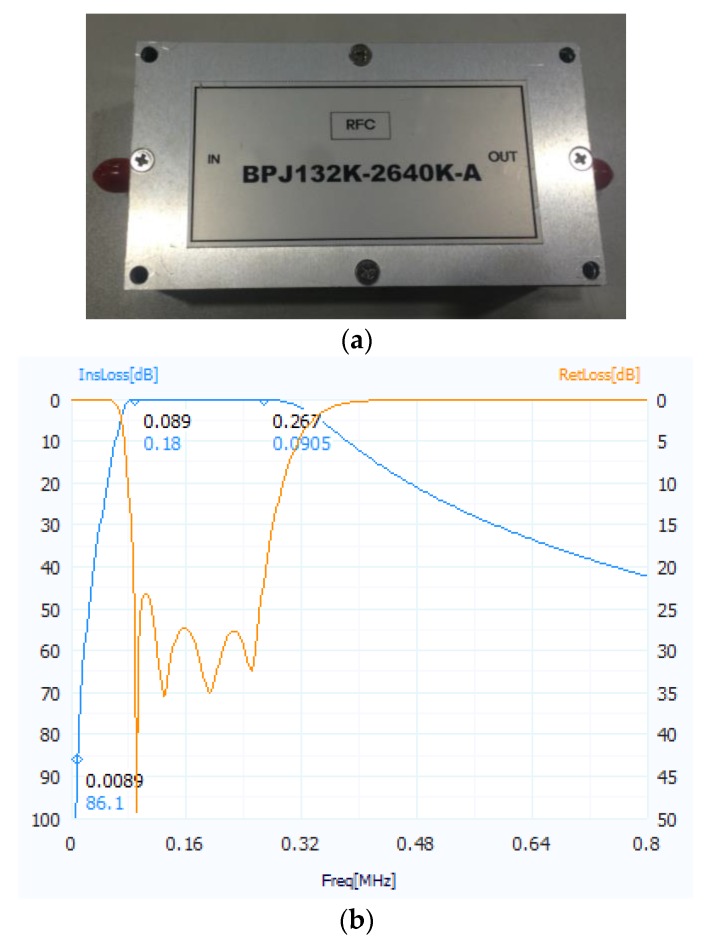
Bandpass filter (**a**) Image of real product (**b**) Filter response.

The very significant application in our TWRI system is the implementation of a high-pass filter. In our implemented system, we use one bandpass filter with a 132 KHz to 2640 KHz BP, as shown in Figure 6a, instead of the conceptual high-pass filter shown in Figure 2. Due to the response of the band-pass filter shown in Figure 6b, this filter can filter the frequency component mainly induced by the concrete wall. As mentioned previously, we concentrate on the imaging of a target behind a wall. Although this BP filter is sufficient for frequency component rejection due to the wall clutter, we applied one subspace projection approach in [29] to our real experiments to mitigate the remaining and dominant frequency components due to wall clutter.

**Figure 7:**
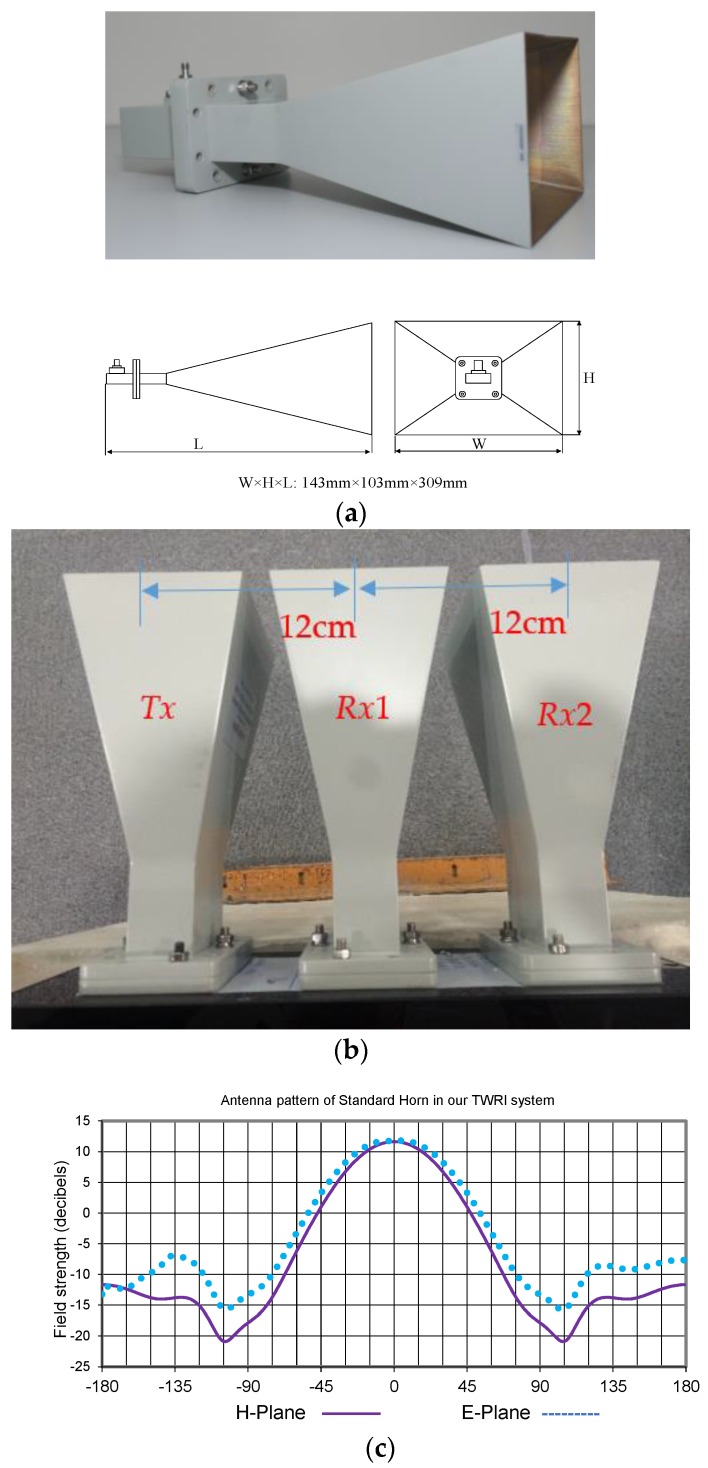
(**a**) Standard horn antenna. (**b**) Antenna array. (**c**) Pattern at 3 GHz, H-plane with 3 dB beam width 43.51° and E-plane pattern with 3 dB beam width 51.32°.

Three identical standard horn antennas with 10 dB gain were employed in our implemented system, one for *Tx* (Back-end) and the others for *Rx*1 and *Rx*2 (Front-end), respectively. The outline and beam patterns of the standard horn antennas are illustrated in Figure 7a. The three centers of the antennas are assigned with equivalent distances of 12 cm, as show in Figure 7b.

**Figure 8:**
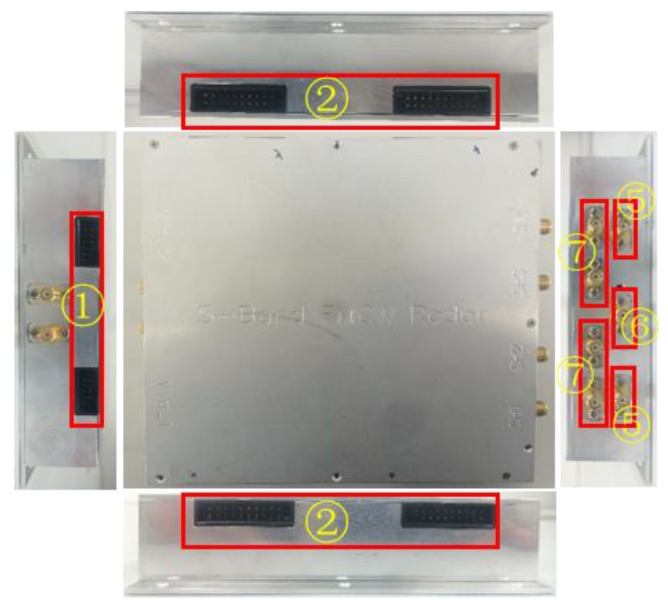
Outward appearance of implemented S-band FMCW TWRI radar system.

**Figure 9:**
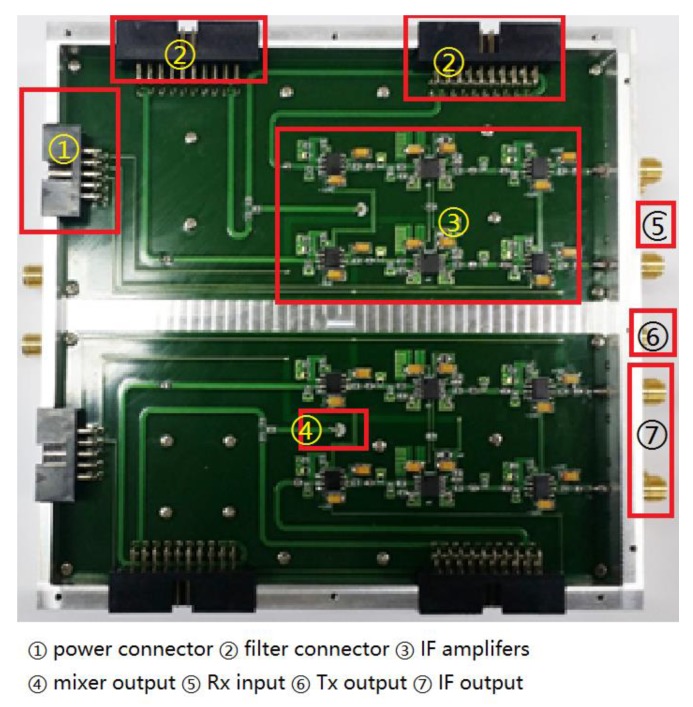
Inward appearance of implemented S-band FMCW TWRI radar system.

The distance between two *Rx* antennas approximately equaled to *λ*, resulting in a beamwidth covering the range from −30° to 30°, which matches the E-plane pattern (51.32°) of standard horn antenna shown in Figure 7c. Figure 7c shows the measured radiation pattern on the E plane at 24 GHz of the transmitting antenna and a receiving antenna in the anechoic chamber. The radiation pattern of the used standard horn antenna is shown in Figure 7c, having HPBW of 92° between 42.6° and 330° and an antenna gain of 11.6 dBi. The implementation of our S-Band FMCW TWRI radar system is shown in Figure 8 and Figure 9 based on the block diagram of Figure 5.

**Figure 10:**
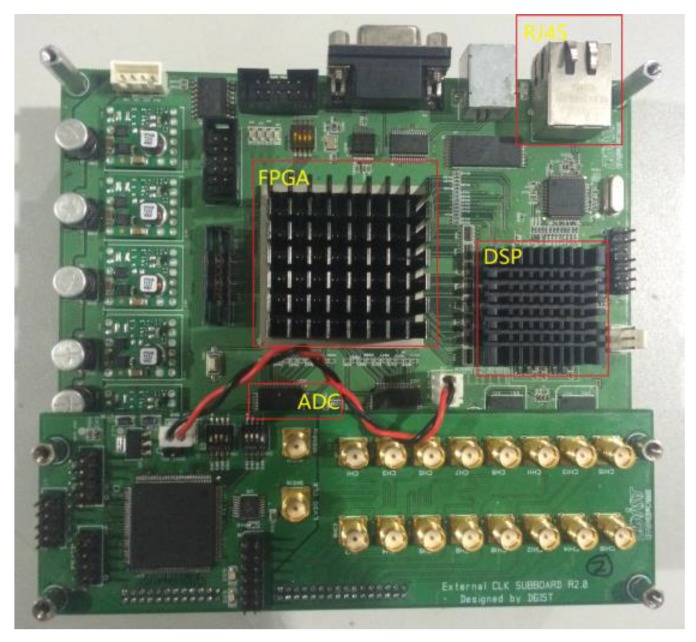
FPGA and DSP board.

The received beat signal was sampled through an FPGA and a DSP board (see Figure 10). The ADC in the implemented radar system has a 12-bit output, and a 12.5 MHz sampling rate. Given that the rule of thumb is that every bit of an ADC represents 6 dB of a dynamic range, the 12-bit ADC provides a 72-dB dynamic range.

After taking system/subsystem issues into account, the specifications of our implemented system are summarized in Table 1.

### 5.2. Experiment Results

Experiments were conducted to test the developed TWRI system. Our FPGA-DSP board is configured to have a 2048-points sample number and a sampling rate of 12.5 MHz. The results were produced by the MATLAB implementation for a particular test data set sampled by the FPGA and DSP board.

**Figure 11:**
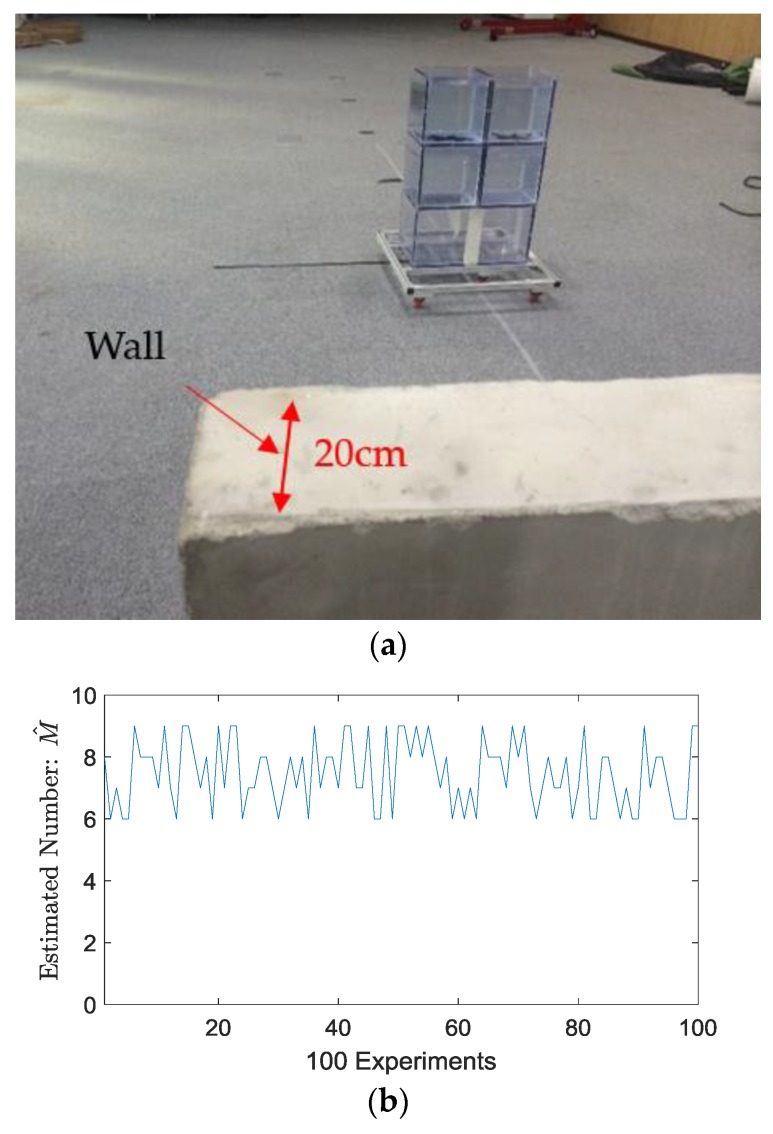

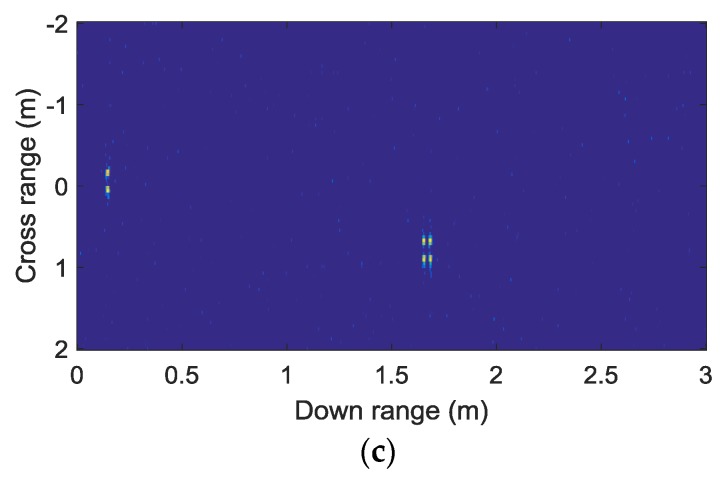
(**a**) Imaged indoor scene, (**b**) Value of M obtained by MDL through 100 experiment trials; (**c**) One frame of the detection results obtained when $\hat{M}$ = 6.

Firstly, an indoor experiment was conducted on a concrete wall inside an office, which was 20 cm thick and with random iron bars inside, as shown in Figure 11a. As previously mentioned, we obtain the number of objects, namely, the value of *M*, through the algorithm MDL. The estimated values of *M* by 100 repeated detection trials are shown in Figure 11b. The water target, four identical square blocks, each with 20 cm side lengths, are arranged in the position 2 m away from the wall with azimuth 30°. One frame of the detection results is shown in Figure 11c, which estimates $\hat{M}$ = 5 for water blocks due to large RCS, about 1 m crossrange, and about 1.7 m downrange. Two significant peaks appeared in the 20 cm-range, 0°~3° azimuth angle due to the wall, which can be mitigated or removed according to the previous works in [25,28,29].

**Figure 12:**
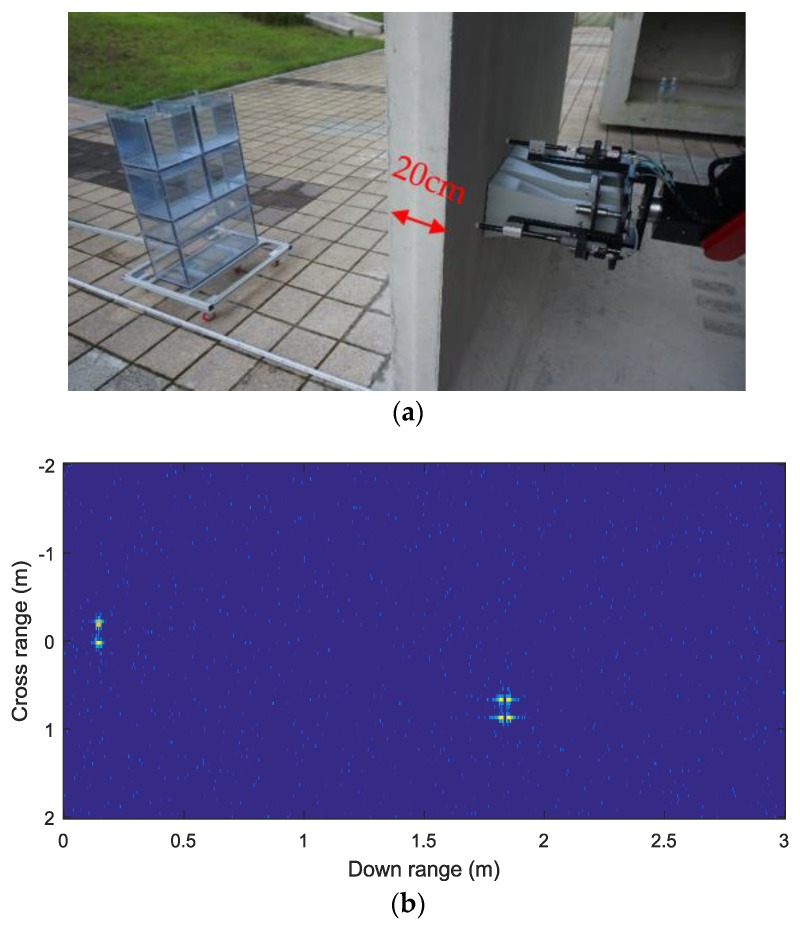

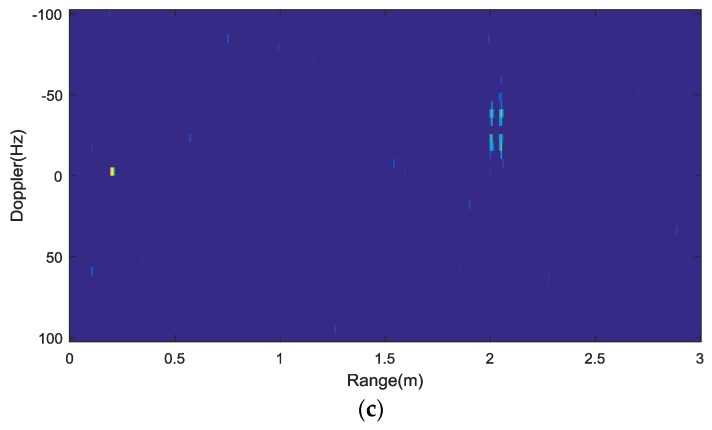
(**a**) Imaged outdoor scene; water target and TWRI system. (**b**) One frame of detection results of multi targets (water blocks) behind the wall when $\hat{M}$ = 5. (**c**) One frame of Range-Doppler maps, showing the Doppler frequency.

A second set of experiments was carried out inside a concrete block behind an office building. This concrete block was specially made for use in an underground sewage system, one side is also 20 cm thick and contains embedded iron bars. Two experiments were organized by detecting the water blocks and an adult man moving away from the radar with a radial speed of 1 m/s, respectively. Figure 12a shows the whole TWRI system where the front-end module is placed in one concrete block, with iron bars inside the wall. The results of detecting the moving water blocks are displayed, and one frame of the results is shown in Figure 12b. The result shown in Figure 12b indicates that the position of peak is crossrange 1 m, and downrange 1.8 m, which means that the azimuth angle is about 29°. The range-Doppler map corresponding to the frame of Figure 12b is shown in Figure 12c which displays the straight-line range about 2.1 m and the Doppler frequency of about −25 Hz of the targets. The wall targets were shown as the zero Doppler components in the range-Doppler map.

**Figure 13:**
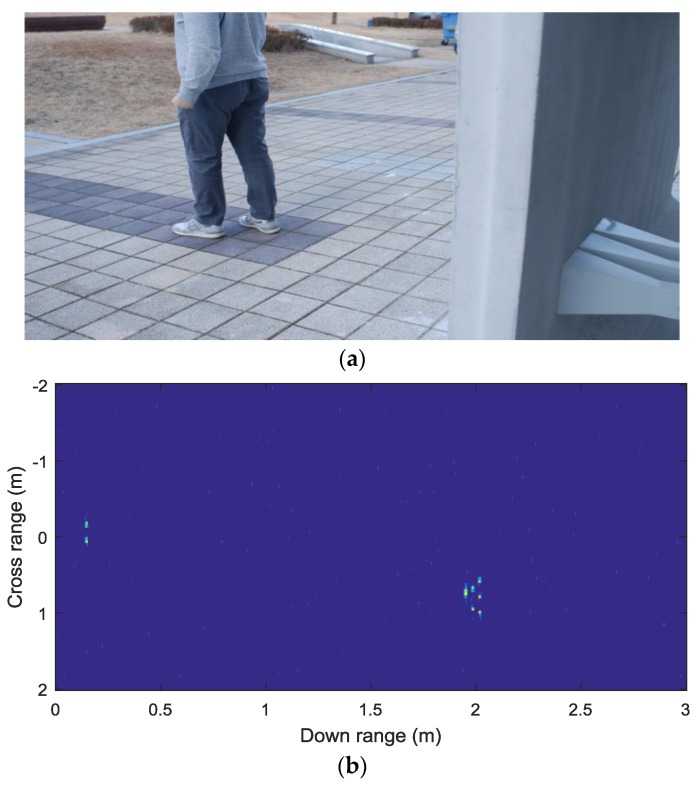

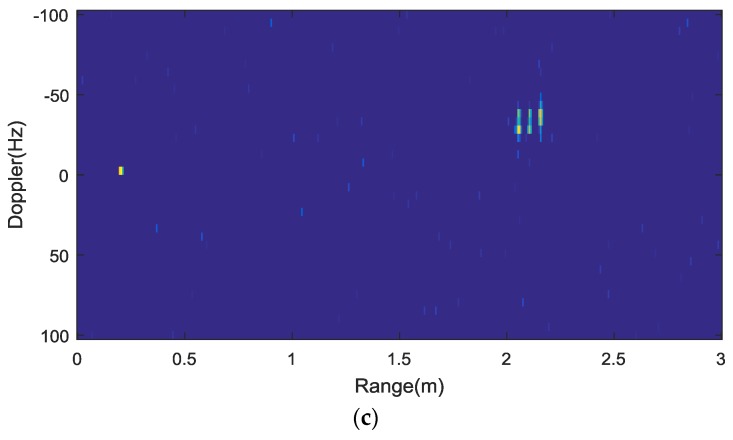
(**a**) Imaged scene for person detection. (**b**) One frame of the detection results when $\hat{M}$ = 8. (**c**) One frame of Range-Doppler maps, showing the Doppler frequency.

For the experiment with a person target, the adult man was moving away from the wall, as shown in Figure 13a. One frame of the detection results is shown in Figure 13b, which estimates $\hat{M}$ = 8 for human body, and the azimuth angle is about 27° with about 1 m crossrange, and about 2 m downrange. Figure 13c shows the corresponding frame of results, which displays the straight-line range about 2.2 m and Doppler frequency of about −25 Hz. The main components of a human, which reflect incoming RF signals, are head, arms, body, and legs as reported in [16]. Thus, it can be observed that more than five peaks were obtained in Figure 13b, corresponding to the main components of humans. Since the two receiving channels are placed in a horizontal direction, the elevation angle of human components is not considered in the suggested TWRI system, resulting in the peak pattern not like that of a human shown in Figure 13b,c.

## 6. Conclusions

This paper has presented a dual channel S-Band FMCW system for through-wall radar imaging and proposed the range-angle-Doppler 3D estimation algorithm based on the 3D shift invariant structure. Our experimental results demonstrated that the implemented TWRI system with the proposed algorithm achieved high quality when imaging targets behind concrete walls. However, the proposed algorithm, comprising a variety of matrix operations such as SVD, is not implemented in FPGA and DSP but rather operated in MATLAB on a PC due to the large operating costs. A study on a low complexity version of the suggested algorithm would be needed to implement a real-time TWRI system with two receiving channels.

## Figures and Tables

**Table 1:** Summary of System Specifications.

Parameter	Specification
Modulation type	FMCW
Receiver	I & Q demodulation
Receiver dynamic range	72 dB
Carrier frequency	2.9 GHz
Bandwidth	600 MHz
Peak power	1 W
Velocity resolution	25 Hz
Range resolution	25 cm
*Tx* and *Rx* antenna	Standard horn antenna
